# Machine-learning prediction of high implantable collamer lens vault using angle-to-angle anatomy: a patient-grouped internal validation study

**DOI:** 10.3389/fcell.2026.1970640

**Published:** 2026-11-03

**Authors:** Hui Li, Runzhang He, Sheng Zhou, Jing Yang

**Affiliations:** 1 Tianjin Key Laboratory of Retinal Functions and Diseases, Tianjin Branch of National Clinical Research Center for Ocular Disease, Eye Institute and School of Optometry, Tianjin Medical University Eye Hospital, Tianjin, China; 2 State Key Laboratory of Ophthalmology, Zhongshan Ophthalmic Center, Sun Yat-sen University, Guangzhou, China

**Keywords:** artificial intelligence, clinical prediction, implantable collamer lens, machine learning, postoperative vault

## Abstract

**Background:**

Machine learning has increasingly been applied to implantable collamer lens (ICL) vault prediction, but the value of anatomically informed feature representation remains uncertain. We evaluated whether angle-to-angle (ATA)-centered anatomy improves prediction of high and continuous postoperative vault compared with white-to-white (WTW)-centered anatomy.

**Methods:**

This retrospective study included 279 eyes from 152 patients with 1-week postoperative vault measurements. Matched regularized supervised machine-learning models were constructed using either ICL size minus WTW or ICL size minus ATA; an expanded ATA model additionally included crystalline lens rise and nasal and temporal anterior chamber angles. Models were internally validated using patient-grouped nested cross-validation, with fellow eyes kept together throughout validation. High vault (>750 µm) was the primary binary outcome, continuous vault was evaluated as a secondary outcome, and low vault (<250 µm) was exploratory. Confidence intervals and between-model differences were estimated using patient-cluster bootstrap sampling.

**Results:**

High vault occurred in 77 eyes (27.6%) from 49 patients. The ATA-centered model achieved a higher AUC for high-vault prediction than the WTW-centered model (0.805 [95% CI, 0.738–0.864] vs. 0.743 [95% CI, 0.667–0.814]), with a paired difference of 0.063 (95% CI, 0.011–0.118). For continuous vault, the ATA-centered model had an MAE of 181.3 µm (95% CI, 163.0–201.1), 17.7 µm lower than the WTW-centered model (95% CI, 4.1–31.7). Adding the expanded ATA variables produced little further improvement. The ATA-centered model also showed good discrimination for low vault (AUC, 0.852), although only 18 patients had this outcome. In a separate paired one-eye analysis, the median within-selection ATA-minus-WTW AUC difference for high vault was 0.049.

**Conclusion:**

ATA-based models improved prediction of both high and continuous 1-week ICL vault compared with otherwise matched WTW-based models. When an ICL size has already been selected, ATA-based prediction may help identify eyes that warrant closer review of biometric measurements and the proposed sizing plan.

## Introduction

Posterior chamber phakic implantable collamer lenses (ICLs) are widely used to correct moderate-to-high myopia while preserving accommodation and the natural crystalline lens. Postoperative vault, defined as the distance between the posterior surface of the ICL and the anterior surface of the crystalline lens, remains an important consideration after implantation. Low vault has been associated with anterior subcapsular cataract, whereas excessive vault may narrow the iridocorneal angle and increase the risk of elevated intraocular pressure or further intervention. Although the central-port design has reduced some complications related to low vault, avoiding both low and high vault remains relevant when selecting ICL size (Chen et al., 2021; Wei et al., 2022; Thompson et al., 2024).

ICL sizing has traditionally relied on horizontal white-to-white (WTW) corneal diameter and anterior chamber depth (ACD) (Seo et al., 2009). WTW is easy to obtain and has long been used in sizing nomograms, but it is an external corneal measurement and does not directly represent the internal space in which the ICL is positioned. Ultrasound biomicroscopy made direct measurement of sulcus-to-sulcus distance possible (Kojima et al., 2012), while swept-source anterior-segment optical coherence tomography (AS-OCT) later provided noncontact measurements of angle-to-angle (ATA) distance and other internal anterior-segment structures (Nakamura et al., 2018; Nakamura et al., 2020; Igarashi et al., 2021). More recent sizing and vault-prediction studies have incorporated ATA, crystalline lens rise (CLR), anterior chamber angle, ciliary-sulcus morphology, and lens-related measurements (Reinstein et al., 2022; Kim et al., 2023; Wu et al., 2023; Yang et al., 2023; Wu et al., 2024). Vault, however, is not determined by ocular width alone. ICL size, crystalline-lens position, pupil size, accommodation, and measurement conditions may also affect the postoperative position of the lens (Gonzalez-Lopez et al., 2018; Thompson et al., 2024).

Beyond ICL surgery, ophthalmic machine learning has also been used for clinical risk prediction, automated quantitative ocular measurements, and forecasting refractive development (Gong et al., 2023; Wan et al., 2023; Zhu et al., 2023). Machine learning (ML), a branch of artificial intelligence (AI), has increasingly been applied to postoperative ICL vault prediction using multivariable biometric data (Kamiya et al., 2021; Russo et al., 2023; Di et al., 2024; Shin et al., 2024; González-Cruces et al., 2025; Li et al., 2026). Comparisons between WTW- and ATA-based approaches remain difficult because published studies differ in ICL type, imaging method, follow-up time, predictor selection, and validation strategy. In clinical AI, predictive performance depends not only on algorithm choice but also on clinically meaningful feature representation and validation design. Recent ophthalmic AI guidance has also emphasized standardized image classification, annotation, and quality control, including for AS-OCT-derived anterior-segment measurements (Yang et al., 2025). Current guidance for prediction models emphasizes transparent reporting, appropriate handling of correlated data, calibration, and validation that reflects the intended use (Collins et al., 2024; Moons et al., 2025). Continuous prediction also gives only part of the clinical picture. High and low vault lie at opposite ends of the distribution and may reflect different anatomical relationships; excessive vault has been linked to ciliary-body morphology, whereas low vault has been associated with iris and posterior-chamber features (Chen et al., 2020; Kato et al., 2020; Khan et al., 2022). Bilateral data introduce another issue. Fellow eyes share substantial anatomical similarity, so separating the two eyes of one patient between model development and validation may lead to overly optimistic performance estimates (Armstrong, 2013; Ying et al., 2018).

Recent work has moved beyond WTW-based sizing by incorporating internal anterior-segment measurements and more comprehensive biometry in direct metric comparisons, vault-optimization studies, analyses of suboptimal vault, and updated prediction formulas (Zhu et al., 2024; Lan et al., 2025; Yang et al., 2026; Tsunehiro et al., 2026). These studies support the relevance of internal anatomy, but they were designed for different objectives and used different predictor combinations, formulas, and validation procedures. Consequently, they do not isolate the incremental effect of replacing a WTW-based anatomical-fit term with an ATA-based term within an otherwise unchanged prediction model.

We therefore evaluated an interpretable ML framework using two otherwise matched models. The models included the same set of predictors, except that one used ICL size minus WTW and the other used ICL size minus ATA. Patient-grouped nested cross-validation was used to ensure that fellow eyes from the same patient remained in the same fold throughout model development and evaluation. The primary objective was to determine whether replacing ICL size minus WTW with ICL size minus ATA improved prediction of high vault at 1 week. Continuous vault was evaluated as a secondary outcome, while low vault was analyzed separately as an exploratory outcome.

## Materials and methods

### Study design and participants

We retrospectively included 279 eyes of 152 patients who underwent toric or nontoric EVO Implantable Collamer Lens implantation (ICL; STAAR Surgical, Monrovia, CA, USA) for myopia at Zhongshan Ophthalmic Center, Sun Yat-sen University, between May 2021 and April 2022. Eligible eyes had a preoperative corneal endothelial cell density >2000 cells/mm^2^, an ACD >2.8 mm, and a central vault measurement at 1 week. We excluded eyes with cataract, glaucoma, corneal dystrophy, or another ocular disorder likely to affect anterior-segment measurements. Patients with diabetes, autoimmune disease, or another relevant systemic disorder were also excluded.

The present study used a cohort from the same center and recruitment period as our previous report (Yang et al., 2023), with partial sample overlap. Specifically, 54 patients (104 eyes) were included in both studies, whereas 98 patients (175 eyes) were unique to the present analysis. In addition, the present cohort was restricted to procedures performed by a single surgeon (J.Y.), unlike the earlier two-surgeon cohort, and differed in biometric distribution, including a higher mean WTW (11.79 ± 0.51 vs. 11.42 ± 0.37 mm).

ICL size was selected with the manufacturer’s Online Calculation and Ordering System (OCOS; STAAR Surgical) using horizontal WTW and ACD. The cohort included 140 toric and 139 nontoric ICLs. All procedures were performed by one surgeon (J.Y.). Procedures were performed through a 3.2-mm corneal incision. After injection of viscoelastic material, the ICL was inserted into the anterior chamber and the haptics were positioned behind the iris. The viscoelastic material was then removed. Nontoric ICLs were placed horizontally, whereas toric ICLs were rotated to the preoperative planned axis.

The study followed the Declaration of Helsinki and was approved by the Ethics Committee of Zhongshan Ophthalmic Center, Sun Yat-sen University (approval no. 2023KYPJ084). The requirement for written informed consent was waived by the Ethics Committee because of the retrospective nature of the study.

### Clinical measurements and outcomes

Preoperative variables included age, sex, eye laterality (right or left), axial length (AL), horizontal WTW, spherical equivalent (SE), intended ICL spherical-equivalent power, ACD, horizontal angle-to-angle (ATA) distance, CLR, temporal and nasal anterior chamber angles, pupil diameter, and selected ICL size. Corneal tomography was obtained with the Pentacam system (OCULUS Optikgeräte GmbH, Wetzlar, Germany), AL with the IOLMaster 700 (Carl Zeiss Meditec AG, Jena, Germany), and WTW by manual slit-lamp caliper measurement. A swept-source AS-OCT system (VG200D; SVision Imaging Ltd., Luoyang, China) was used to measure ACD, ATA, CLR, pupil diameter, anterior chamber angles, and postoperative vault.

One operator acquired pupil-centered AS-OCT scans along the horizontal meridian. Three measurements were taken and averaged. ACD was the distance from the posterior corneal surface to the anterior crystalline lens surface; ATA was the distance between the nasal and temporal angle recesses; and CLR was the perpendicular distance from the anterior crystalline lens surface to the line joining the angle recesses. Anatomical fit for the candidate ICL size was expressed as ICL size minus WTW and ICL size minus ATA. All preoperative biometric parameters and postoperative vault measurements were obtained three times and averaged for analysis. Illumination, fixation, and accommodation conditions were not standardized or documented for the preoperative or postoperative examinations.

Central vault was measured with the same AS-OCT protocol 1 week after surgery and defined as the distance between the posterior ICL surface and the anterior crystalline lens surface at the lens center. High vault (>750 µm) was the primary binary outcome. Continuous 1-week vault was also evaluated, and low vault (<250 µm) was analyzed separately as an exploratory outcome. These thresholds were used for analysis and were not intended as universal intervention criteria.

### Machine-learning model development and validation

The WTW-centered predictor set included age, sex, eye laterality, AL, SE, intended ICL spherical-equivalent power, ACD, pupil diameter, selected ICL size, and ICL size minus WTW. The ATA-centered set replaced ICL size minus WTW with ICL size minus ATA, while the expanded ATA model additionally included CLR and the temporal and nasal anterior chamber angles. These candidate predictors were selected based on variables previously reported to be associated with postoperative vault and were restricted to measurements available in the retrospective dataset. Other potentially relevant biometric parameters, such as sulcus-to-sulcus distance, crystalline lens thickness, and additional posterior-chamber or ciliary-body measurements, were not systematically recorded and therefore could not be evaluated.

Missing predictors were imputed using training-set medians, and all predictors were standardized before model fitting. Sex was coded as female = 0 and male = 1. Sex was unavailable in the existing research dataset for 11 patients; these patients remained classified as missing in descriptive summaries. For modeling, median imputation was fitted within each training fold; because the observed training-fold median was 0, missing sex values received the female-coded value without being reclassified as female for descriptive purposes. A separate sensitivity analysis repeated the validation after assigning all 11 missing values to the male-coded value. Ridge regression and L2-regularized logistic regression were used as supervised machine-learning models for continuous vault and binary high- and low-vault outcomes, respectively. The regularized framework was chosen to keep the ATA-versus-WTW comparison interpretable while limiting instability from correlated clinical and biometric predictors. Internal validation used patient-grouped nested cross-validation, with both eyes from the same patient kept in the same fold (Steyerberg et al., 2001). Performance was based on out-of-fold predictions.

For the primary analysis, nested five-fold cross-validation was repeated 10 times, with patients grouped in both the outer and inner folds. Within each outer training set, four-fold patient-grouped inner validation selected the regularization parameter by AUC for binary high- and low-vault prediction and by MAE for continuous-vault prediction; binary splits were stratified. Imputation and standardization were fitted using training data only at each validation stage. Each eye received one held-out prediction in each outer repetition, and the 10 held-out predictions were averaged before performance metrics were calculated.

After validation, the ATA-centered continuous-vault and high-vault models were refitted to the full cohort to provide final equations. No final low-vault equation was provided because only 18 patients had low vault. Complete model parameters are provided in Supplementary Table S1.

### Sensitivity analyses

Two sensitivity analyses were performed. First, to assess the influence of missing sex information, model validation was repeated after assigning all 11 patients with missing sex values to the male-coded value.

Second, to assess the influence of including both eyes from the same patient, we performed a paired one-eye analysis using 100 repeated random selections of one eye per patient (152 eyes per selection). Within each selection, the ATA- and WTW-based models were evaluated using the same eyes and identical five-fold validation assignments. Unlike the primary analysis, fixed regularization parameters were used without nested tuning. Results were summarized as median model performance and median within-selection paired differences between ATA and WTW, with interquartile ranges (IQRs) across the 100 selections.

### Statistical analysis

Model performance was assessed using MAE, RMSE, and R^2^ for continuous vault, and AUC, average precision, and Brier score for high- and low-vault classification. Calibration was also examined (Van Calster et al., 2019). Ninety-five percent confidence intervals and paired between-model differences were estimated using 1,500 patient-cluster bootstrap resamples, with all eligible eyes from each sampled patient retained. Bootstrap estimates were calculated from the averaged out-of-fold predictions without model refitting. For the high-vault model, an illustrative operating point at a predicted risk of 25% was additionally summarized. This threshold was selected *post hoc* for illustrative purposes.

Fellow-eye association was assessed using Pearson correlation and the intraclass correlation coefficient, while inter-eye agreement was summarized by the absolute inter-eye difference and Bland–Altman limits of agreement. Sensitivity analyses were evaluated using the same performance metrics as the primary analysis; repeated one-eye results were summarized using medians and interquartile ranges across the 100 selections. Analyses were performed in Python version 3.12.13, with reporting guided by TRIPOD + AI and PROBAST + AI recommendations (Collins et al., 2024; Moons et al., 2025).

## Results

### Cohort characteristics

The study included 279 eyes from 152 patients, of whom 127 contributed both eyes and 25 contributed one eye. Mean 1-week vault was 607.7 ± 296.4 µm. High vault occurred in 77 eyes (27.6%) from 49 patients, and low vault in 26 eyes (9.3%) from 18 patients (Figure 1A). Cohort characteristics are summarized in Table 1.

**FIGURE 1:**
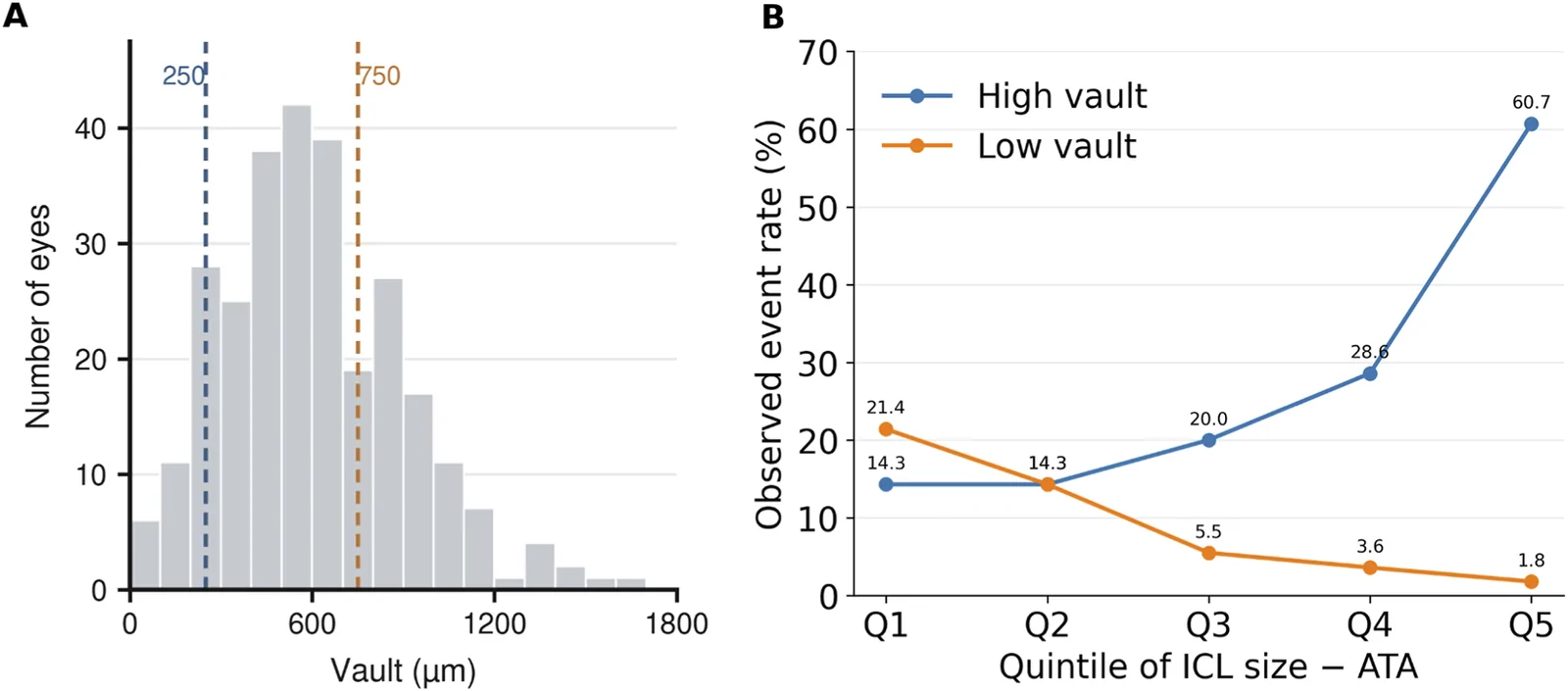
Distribution of 1-week vault and anatomical gradient. **(A)** Distribution of central vault at 1 week, with thresholds of 250 and 750 µm. **(B)** Observed rates of high vault (>750 µm) and low vault (<250 µm) across quintiles of ICL size minus ATA.

**TABLE 1:** Cohort characteristics.

Characteristic	Overall cohort
Patients, n	152
Eyes, n	279
Bilateral/unilateral patients, n	127/25
Age, years	27.7 ± 6.5
Female/male/missing sex, patients, n	93/48/11
Right/left eyes, n	139/140
Axial length, mm	27.87 ± 2.05
WTW, mm	11.79 ± 0.51
Manifest spherical equivalent, D	−11.24 ± 4.48
ICL spherical-equivalent power, D	−12.31 ± 3.85
Anterior chamber depth, mm	3.25 ± 0.21
ATA, mm	11.83 ± 0.41
Crystalline lens rise, µm	−35.2 (−155.2 to 148.0)
Temporal/nasal ACA, degrees	52.17 ± 14.41/52.17 ± 14.35
Pupil diameter, mm	4.50 ± 0.90
ICL size, mm	12.60 ± 0.41
ICL size distribution (12.1/12.6/13.2/13.7 mm), n	79/135/62/3
ICL size − WTW, mm	0.82 ± 0.35
ICL size − ATA, mm	0.77 ± 0.33
Vault at 1 week, µm	607.7 ± 296.4; median 585.2 (400.4–802.4)

Values are mean ± standard deviation or median (interquartile range) unless otherwise stated. Patient-level age and sex are reported once per patient; ocular measurements are eye-level. ACA = anterior chamber angle; ATA = angle-to-angle; ICL = implantable collamer lens; WTW = white-to-white.

Among the 127 patients with bilateral data, vault was strongly correlated between fellow eyes (Pearson r = 0.886; ICC = 0.877). However, the median absolute inter-eye difference was 72.8 µm (IQR, 31.8–151.5 µm), and the 95% limits of agreement for the right-minus-left difference were −251.6 to 320.4 µm, indicating appreciable inter-eye variation.

### High-vault prediction

The ATA-centered predictor set showed better discrimination for high vault than the WTW-centered set, with AUCs of 0.805 (95% CI, 0.738–0.864) and 0.743 (95% CI, 0.667–0.814), respectively. The paired AUC improvement was 0.063 (95% CI, 0.011–0.118). Average precision and Brier score also favored ATA. Adding CLR and anterior chamber angles produced little further improvement (AUC, 0.812; difference vs. the simpler ATA set, 0.007; 95% CI, −0.024–0.035) (Figure 2B; Table 2).

**FIGURE 2:**
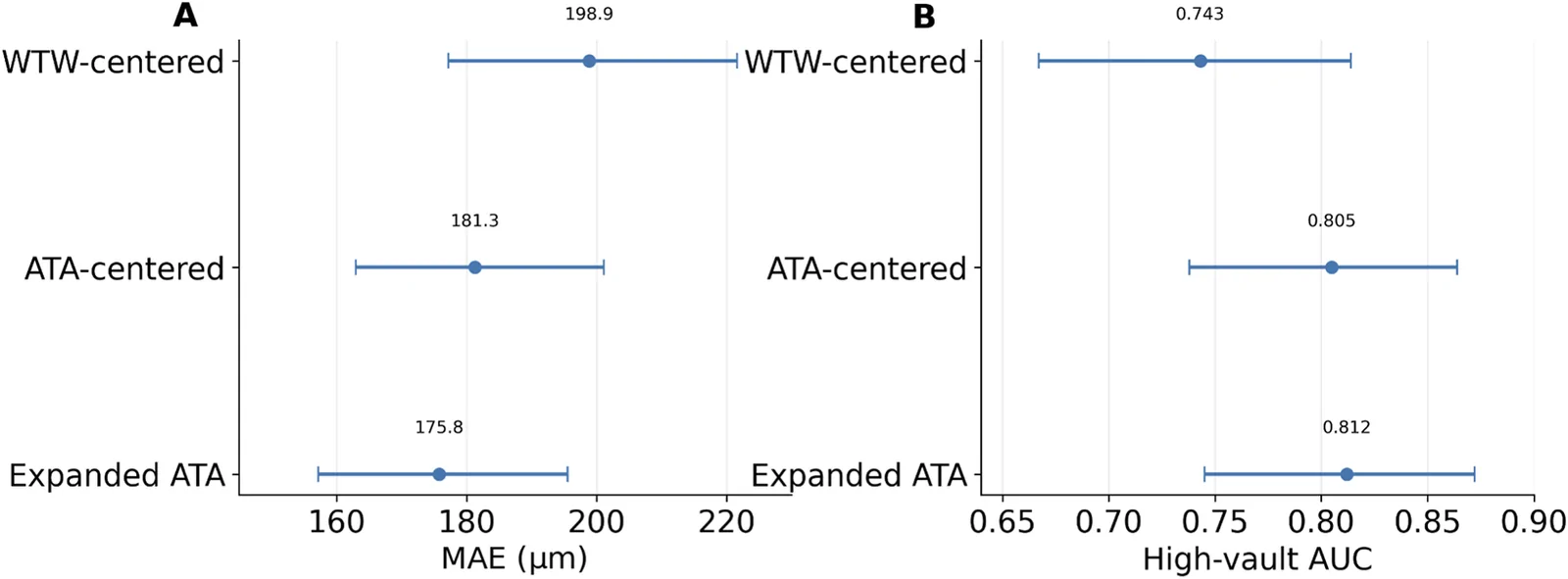
Internally validated performance across the WTW-centered, ATA-centered, and expanded ATA predictor sets. **(A)** Mean absolute error (MAE) for continuous 1-week vault prediction. **(B)** Area under the receiver operating characteristic curve (AUC) for high-vault (>750 µm) prediction. Error bars indicate patient-cluster bootstrap 95% confidence intervals.

**TABLE 2:** Internally validated performance across the WTW-centered, ATA-centered, and expanded ATA predictor sets.

Outcome	Predictor set	Key metric	Other metrics
Continuous vault	WTW-centered	MAE, 198.9 µm (95% CI, 177.2–221.6)	RMSE, 249.8 µm; R^2^, 0.287
​	ATA-centered	MAE, 181.3 µm (95% CI, 163.0–201.1)	RMSE, 225.2 µm; R^2^, 0.421
​	Expanded ATA	MAE, 175.8 µm (95% CI, 157.2–195.6)	RMSE, 222.7 µm; R^2^, 0.433
High vault (>750 µm)	WTW-centered	AUC, 0.743 (95% CI, 0.667–0.814)	AP, 0.497; Brier score, 0.173
​	ATA-centered	AUC, 0.805 (95% CI, 0.738–0.864)	AP, 0.611; Brier score, 0.157
​	Expanded ATA	AUC, 0.812 (95% CI, 0.745–0.872)	AP, 0.622; Brier score, 0.151
Low vault (<250 µm)	WTW-centered	AUC, 0.801 (95% CI, 0.695–0.889)	AP, 0.261; Brier score, 0.077
​	ATA-centered	AUC, 0.852 (95% CI, 0.766–0.919)	AP, 0.351; Brier score, 0.072
​	Expanded ATA	AUC, 0.852 (95% CI, 0.772–0.917)	AP, 0.345; Brier score, 0.073

MAE was used as the key performance metric for continuous vault and AUC, for high- and low-vault classification. AP = average precision; ATA = angle-to-angle; AUC = area under the receiver operating characteristic curve; CI = confidence interval; MAE = mean absolute error; RMSE = root mean square error; WTW = white-to-white.

The calibration intercept and slope of the ATA-centered high-vault model were 0.345 and 1.456, respectively. Grouped calibration showed over prediction at low predicted risk and under prediction at high predicted risk (Figure 3).

**FIGURE 3:**
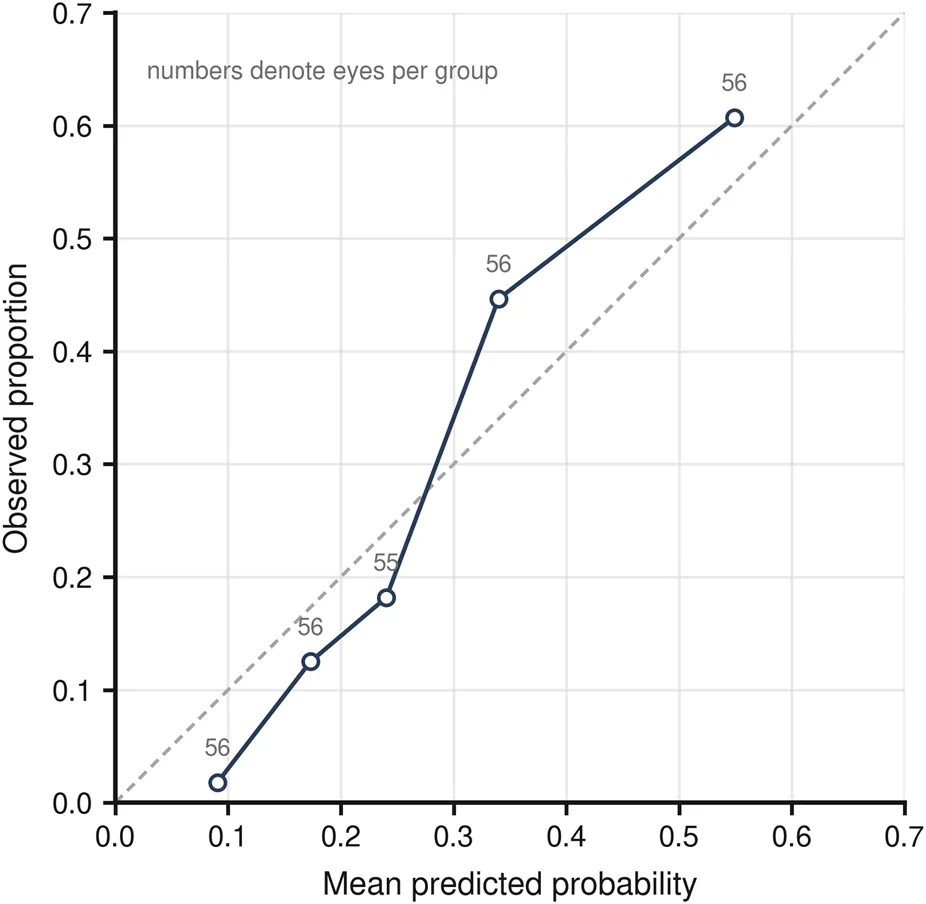
Calibration of the ATA-centered high-vault model. Observed high-vault rates are plotted against mean predicted probabilities across quintiles of predicted risk.

At the *post hoc* illustrative 25% predicted-risk threshold, 133 of 279 eyes were flagged for additional review. Among 77 high-vault eyes, 65 were identified and 12 were missed, corresponding to sensitivity 84.4%, specificity 66.3%, positive predictive value 48.9%, and negative predictive value 91.8%. Exploratory decision-curve analysis showed that the ATA-centered model provided greater net benefit than the review-all and review-none strategies across the evaluated threshold probabilities of 10%–40%. At the 25% threshold, the net benefit was 0.152 for the ATA-centered model, compared with 0.035 for the review-all strategy and 0 for the review-none strategy (Supplementary Figure S1).

### Continuous-vault prediction

The ATA-centered predictor set also performed better for continuous vault prediction, with an MAE of 181.3 µm (95% CI, 163.0–201.1 µm) compared with 198.9 µm (95% CI, 177.2–221.6 µm) for WTW. The corresponding MAE reduction was 17.7 µm (95% CI, 4.1–31.7 µm), with higher R^2^ for ATA than WTW (0.421 vs. 0.287). The expanded ATA set provided only a small additional reduction in MAE (5.6 µm; 95% CI, −0.2–11.2 µm) (Figure 2A; Table 2).

### Low-vault prediction

In this exploratory analysis, low-vault discrimination was numerically higher with the ATA-centered predictor set than with WTW (AUC, 0.852 vs. 0.801), although the paired difference was imprecise (0.052; 95% CI, −0.034–0.138). Because low vault occurred in only 18 patients, this result should be interpreted cautiously (Table 2).

### Anatomical gradient and sensitivity analysis

A clear anatomical gradient was seen across quintiles of ICL size minus ATA. High-vault prevalence increased from 14.3% in the lowest quintile to 60.7% in the highest, whereas low-vault prevalence decreased from 21.4% to 1.8% (Figure 1B). In the ATA-centered models, ACD and pupil diameter were positively associated with continuous and high vault, while age was negatively associated with high vault.

In the paired one-eye sensitivity analysis, median high-vault AUC was 0.787 (IQR, 0.774–0.807) for the ATA-based model and 0.740 (IQR, 0.721–0.752) for the WTW-based model. The median within-selection ATA-minus-WTW AUC difference was 0.049 (IQR, 0.043–0.059). For continuous vault, median MAE was 179.6 µm (IQR, 174.8–183.9) for ATA and 195.2 µm (IQR, 192.0–198.2) for WTW, corresponding to a median WTW-minus-ATA MAE reduction of 16.3 µm (IQR, 13.8–18.3). The paired differences favored ATA for both outcomes in all 100 one-eye selections (Supplementary Figure S2).

In the missing-sex sensitivity analysis, assigning all 11 missing sex values to the male-coded value yielded high-vault AUCs of 0.796 for ATA and 0.746 for WTW. For continuous vault, the corresponding MAEs were 182.0 µm for ATA and 200.7 µm for WTW. These results were consistent with the primary analysis and did not alter the direction of the ATA-versus-WTW difference.

Final coefficients and implementation details for the ATA-centered continuous- and high-vault models are provided in Supplementary Table S1.

## Discussion

Replacing WTW with ATA improved prediction of both high vault and continuous 1-week vault when the other predictors were kept unchanged. The AUC for high vault increased from 0.743 to 0.805, and the MAE for continuous vault decreased by 17.7 µm. By comparison, adding CLR and the nasal and temporal anterior chamber angles resulted in only small additional gains. ATA also performed numerically better for low vault, although this part of the analysis was based on relatively few events. Overall, these findings suggest that the choice of biometric representation contributed more to model performance than simply expanding the number of predictors.

The difference between ATA and WTW is not unexpected from an anatomical point of view. WTW is an external corneal measurement and has been widely used in ICL sizing because it is easy to obtain, but it does not directly describe the internal width of the anterior segment. ATA is measured closer to the space occupied by the ICL and may therefore better reflect the relationship between the selected ICL size and internal ocular dimensions (Seo et al., 2009; Kojima et al., 2012; Nakamura et al., 2018; Nakamura et al., 2020; Igarashi et al., 2021; Reinstein et al., 2022; Kim et al., 2023; Wu et al., 2023; Yang et al., 2023; Wu et al., 2024). In our data, larger values of ICL size minus ATA showed the clearest relationship with postoperative vault. Larger values were associated with greater vault and a higher frequency of high vault, whereas low vault became less common. The same trend was seen across quintiles of ICL size minus ATA, providing a more intuitive clinical representation of this relationship.

Recent studies have similarly emphasized the value of internal anterior-segment measurements in ICL sizing and vault prediction. Zhu et al. (2024) compared WTW, ATA, and sulcus-to-sulcus measurements for postoperative vault prediction, while Lan et al. (2025), Yang et al. (2026), and Tsunehiro et al. (2026) incorporated ATA and other internal biometric parameters into sizing or vault-prediction approaches. These studies support the relevance of internal ocular anatomy, but they have generally focused on developing or refining formulas, identifying multiple predictive factors, or comparing broader sizing strategies. The present study addressed a more specific question by comparing two otherwise matched models in which the principal difference was the use of ICL size minus WTW versus ICL size minus ATA. This design allowed the incremental predictive contribution associated with replacing WTW with ATA to be evaluated more directly.

The observed advantage of ATA may reflect both its closer anatomical relationship to the internal anterior segment and differences in measurement methodology. In this study, WTW was measured manually with a slit-lamp caliper, whereas ATA was obtained using swept-source AS-OCT. Because these measurements were acquired using different techniques, measurement precision may have contributed to the observed performance difference. Future studies using more comparable acquisition methods could help further clarify the relative contribution of anatomical location and measurement technique.

Vault was not determined by ATA alone. ACD and pupil diameter were positively associated with continuous and high vault, while age was negatively associated with high vault. A deeper anterior chamber may partly reflect differences in the overall anterior-segment configuration and crystalline-lens position. Pupil diameter is more variable and is affected by illumination and accommodation, both of which can also change anterior-segment geometry and measured vault (Gonzalez-Lopez et al., 2018; Chen et al., 2020; Kato et al., 2020; Khan et al., 2022). This is relevant in practice because pupil-related measurements obtained under different lighting conditions may not be directly comparable. The association with age is less easy to explain. It may reflect age-related changes in accommodation, lens position, or other biometric characteristics that were not fully represented by the measured variables. These associations are therefore more useful as part of the overall anatomical picture than as isolated determinants of vault.

There are also important structures that were not measured directly in this study. The ICL ultimately rests in the posterior chamber, and ciliary-sulcus morphology, haptic position, ICL orientation, and crystalline-lens contour can all affect its final position (Gonzalez-Lopez et al., 2018; Chen et al., 2020; Kato et al., 2020; Wei et al., 2022; Khan et al., 2022; Thompson et al., 2024). These factors may account for part of the remaining prediction error, especially in eyes with very high or very low vault. ATA is therefore better viewed as a more relevant width measurement than WTW, not as a complete description of the space in which the ICL rests.

The expanded ATA model did not clearly improve on the simpler model. This does not mean that CLR or anterior chamber angle measurements are unimportant. Some of these variables are correlated and may provide overlapping anatomical information. With only 152 patients, weaker effects or interactions between several measurements may also be difficult to identify reliably. More detailed posterior-chamber imaging could improve prediction in a larger cohort, but the present data did not show a clear advantage from adding more anterior-segment variables.

From a machine-learning perspective, the main improvement came from feature representation rather than greater model complexity. Replacing WTW with ATA improved performance within otherwise matched regularized models, whereas adding CLR and chamber-angle measurements produced little incremental gain. The validation strategy was also important because fellow-eye vaults were strongly correlated. Both eyes were therefore retained while ensuring that fellow eyes from the same patient remained in the same validation fold. In the paired one-eye sensitivity analysis, ATA and WTW were evaluated on the same selected eyes and validation assignments, and the paired differences favored ATA for both high-vault discrimination and continuous-vault prediction in all 100 selections. Results were also stable when the 11 missing sex values were assigned to the male-coded value, indicating that the direction of the ATA-versus-WTW comparison was not materially affected by either bilateral inclusion or the handling of missing sex information.

The performance of the model also needs to be considered in terms of how it might actually be used. The high-vault model separated higher- and lower-risk eyes reasonably well, but the predicted probabilities were not well calibrated. The continuous model also retained substantial prediction error. For this reason, the model is more suitable for identifying eyes that may need additional review than for giving an exact postoperative vault. At the *post hoc* illustrative 25% predicted-risk threshold, 65 of 77 high-vault eyes were identified, corresponding to a sensitivity of 84.4%, although 133 of 279 eyes would have been flagged for review. Exploratory decision-curve analysis also showed greater net benefit for the ATA-based model than for the review-all and review-none strategies across the evaluated threshold range. A high predicted risk might lead to repeat measurement of ATA or other biometric parameters, a more careful review of the proposed ICL size, or closer early postoperative follow-up. It should not, however, be used on its own to justify choosing a smaller lens.

This point is important because the study does not contain outcomes for alternative ICL sizes in the same eye. Each eye contributed only the size that was actually implanted. The observed associations with ICL size and ICL size minus ATA therefore reflect the sizing decisions made in this cohort. The model can estimate risk within this observed range of practice, but it cannot tell what would have happened if another size had been selected. Accordingly, neither the predicted risk nor the illustrative 25% threshold should be interpreted as a rule for automatically selecting a smaller or larger ICL.

This study has several limitations. First, its retrospective and single-center design may limit the generalizability of the findings. Second, WTW and ATA were obtained using different measurement techniques, and formal comparative repeatability data were unavailable. Illumination, fixation, and accommodation conditions were also not standardized or documented, which may have introduced additional measurement variability. Third, the number of low-vault events was limited, and postoperative vault was assessed only at 1 week; therefore, the findings may not extend to later postoperative changes or to the prediction of low vault. In addition, several potentially relevant anatomical parameters, including sulcus-to-sulcus distance, crystalline lens thickness, ciliary-sulcus morphology, and postoperative haptic position, were not systematically available in this retrospective dataset. Finally, all model performance estimates were derived from internal validation. Independent external validation, including assessment of calibration and potential recalibration across different populations and imaging systems, is required before clinical application.

## Conclusion

In an otherwise matched modeling framework, replacing WTW with ATA improved prediction of both high and continuous 1-week ICL vault, with consistent findings across sensitivity analyses. Incorporating ATA into preoperative assessment may therefore improve identification of eyes in which a clinician-selected ICL size warrants additional scrutiny, particularly through repeat biometric verification or closer postoperative monitoring. These results support ATA as a clinically informative biometric parameter for vault risk assessment and provide a basis for further external validation in broader clinical settings.

## Data Availability

The raw data supporting the conclusions of this article are available from the corresponding authors upon reasonable request and subject to their approval.

## Supplementary material

Supplementary Figure S1 Exploratory decision-curve analysis for high-vault prediction. Net benefit of the ATA-centered model is compared with the review-all and review-none strategies across predicted-risk thresholds of 10%–40%. The dashed vertical line marks the illustrative 25% threshold.

Supplementary Figure S2 Paired one-eye sensitivity analysis across 100 random selections of one eye per patient, with 152 eyes per selection. Panel A shows ATA-minus-WTW differences in high-vault AUC; panel B shows WTW-minus-ATA differences in continuous-vault MAE. Dots represent individual selections; open circles and horizontal bars indicate medians and interquartile ranges. Positive values favor ATA.
