# Supplementary material for: Machine-learning prediction of high implantable collamer lens vault using angle-to-angle anatomy: a patient-grouped internal validation study
Source: Front Cell Dev Biol. 2026 Nov 3;14:1970640. doi: 10.3389/fcell.2026.1970640 (PMC13732134; doi:10.3389/fcell.2026.1970640)
Supplement: Supplementary file 1 [file Supplementaryfile1.docx]

**Table S1. Final specification of the ATA-centered continuous-vault and high-vault models fitted to the full development cohort.**

| **Predictor** | **Imputation median** | **Center (scale)** | **Continuous-vault β** | **High-vault β** |
| --- | --- | --- | --- | --- |
| Age, years | 26.000 | 27.612903 (6.457524) | −18.058170 | −0.188484 |
| Male sex (1=male; 0=female) | 0.000 | 0.315412 (0.464680) | −48.749442 | −0.067129 |
| Right eye (1=right; 0=left) | 0.000 | 0.498208 (0.499997) | 14.354824 | 0.058065 |
| Axial length, mm | 27.470 | 27.874229 (2.044435) | 55.914742 | 0.133875 |
| Manifest spherical equivalent, D | −10.750 | −11.238351 (4.466983) | 36.685524 | −0.033024 |
| ICL spherical-equivalent power, D | −12.250 | −12.308244 (3.844358) | −19.418018 | 0.010421 |
| Anterior chamber depth, mm | 3.263 | 3.254652 (0.213385) | 53.002431 | 0.189978 |
| Pupil diameter, mm | 4.489 | 4.495522 (0.896287) | 52.187811 | 0.278155 |
| Selected ICL size, mm | 12.600 | 12.603584 (0.404705) | 68.534169 | 0.389359 |
| ICL size minus ATA, mm | 0.753 | 0.768833 (0.329418) | 115.989185 | 0.455827 |

For each predictor, calculate z = (x − center)/scale after applying the listed median if the value is missing. Predicted vault (µm) = 607.692229 + Σ(βcontinuous × z). P(high vault) = 1/{1 + exp[−(−1.129679 + Σ(βhigh × z))]}. The final ridge α was 10 and logistic C was 0.03. This full-cohort refit is provided for implementation only; internally validated performance is reported in Table 2 of the main manuscript.
